# Genomic features of *Bordetella parapertussis *clades with distinct host species specificity

**DOI:** 10.1186/gb-2006-7-9-r81

**Published:** 2006-09-06

**Authors:** Mary M Brinig, Karen B Register, Mark R Ackermann, David A Relman

**Affiliations:** 1 Department of Microbiology and Immunology, Stanford University School of Medicine, Stanford, California 94305, USA; 2 VA Palo Alto Health Care System, Palo Alto, California 94304, USA; 3 USDA/ARS/National Animal Disease Center, Respiratory Diseases of Livestock Research Unit, Ames, Iowa 50010, USA; 4 Department of Veterinary Pathology, Iowa State University, Ames, Iowa 50011, USA; 5 Department of Medicine, Stanford University School of Medicine, Stanford, California 94305, USA

## Abstract

**Background:**

The respiratory pathogen *Bordetella parapertussis *is a valuable model in which to study the complex phenotype of host specificity because of its unique two-species host range. One subset of strains, including the sequenced representative, causes whooping cough in humans, while other strains infect only sheep. The disease process in sheep is not well understood, nor are the genetic and transcriptional differences that might provide the basis for host specificity among ovine and human strains.

**Results:**

We found 40 previously unknown genomic regions in an ovine strain of *B. parapertussis *using subtractive hybridization, including unique lipopolysaccharide genes. A microarray survey of the gene contents of 71 human and ovine strains revealed further differences, with 47 regions of difference distinguishing the host-restricted subgroups. In addition, sheep and human strains displayed distinct whole-genome transcript abundance profiles. We developed an animal model in which sheep were inoculated with a sheep strain, human strain, or mixture of the two. We found that the ovine strain persisted in the nasal cavity for 12 to 14 days, while the human strain colonized at lower levels and was no longer detected by 7 days post-inoculation. The ovine strain induced less granulocyte infiltration of the nasal mucosa.

**Conclusion:**

Several factors may play a role in determining host range of *B. parapertussis*. Human- and ovine-associated strains have differences in content and sequence of genes encoding proteins that mediate host-pathogen contact, such as lipopolysaccharide and fimbriae, as well as variation in regulation of toxins, type III secretion genes, and other virulence-associated genes.

## Abstract 2

Genomic analysis of human and ovine *Bordetella parapertussis *strains reveals differences distinguishing the host-restricted subgroups.

## Background

Whooping cough, with its prolonged paroxysmal cough and distinctive 'whoop', was well-known by the Middle Ages [1]. *Bordetella pertussis *was isolated from whooping cough patients in 1906, and the pathogenesis of this disease has been the subject of extensive study. In the 1930s Eldering and Kedrick noticed different colony morphology of some strains, which they used as the basis for proposing a new species, *B. parapertussis *[2]. Later estimates of the percentage of *Bordetella*-associated cases of cough caused by *B. parapertussis *range from about 5% to 30% [3-6], depending on case definition and vaccination coverage. This species was considered an obligate human pathogen until 1987, when *B. parapertussis*-like organisms were found in normal and pneumonic lamb lungs [7,8]. *B. parapertussis *strains from humans appear to be genetically distinct from ovine strains, and while human strains are highly clonal, ovine strains appear more heterogeneous by pulsed-field gel electrophoresis [9], insertion element typing [10], and PCR-based random amplified polymorphic DNA profiles [11]. This species has not been isolated from any other source and is now thought to be composed of two subgroups, one of which infects only humans and the other of which infects only sheep.

The unique host specificity of *B. parapertussis *contrasts with both the obligate human pathogen lifestyle of *B. pertussis *as well as the broad mammalian host range of the third member of this close family of respiratory pathogens, *B. bronchiseptica*. Despite considerable genetic similarity among the three species (or subspecies, as some have proposed), together they illustrate a spectrum of host restriction, and may provide insights into the evolution and maintenance of this important phenotype. *B. parapertussis *affords one of the least-confounded models of host specificity in the bacterial world, because its two subgroups are found in only two hosts, and are known to be very closely related.

While one human-associated strain of *B. parapertussis *has been sequenced completely [12], much less is known about ovine strains, so we undertook a comprehensive investigation of a large collection of such strains, utilizing subtractive hybridization, microarray-based comparative genomic hybridization, and transcript abundance profiling. In addition, we developed an experimental sheep infection model to explore the colonization patterns and resulting pathology caused by an ovine strain of *B. parapertussis*, compared to a human strain. We found that ovine strains contain a large amount of genetic material not found in human strains, including a unique lipopolysaccharide (LPS) locus and an additional gene encoding a fimbrial subunit, and that the two groups of strains have many differences with respect to genome content and transcript abundance profile. Of particular interest were differences in transcript abundances for several virulence-associated genes and operons, including tracheal colonization factor, dermonecrotic toxin, adenylate cyclase, and the *bsc *type III secretion system. Inoculation of sheep with either the natural ovine pathogen or a human strain revealed that the ovine strain elicited a less intense granulocyte infiltrate and colonized the nasal turbinate more efficiently and for a longer period of time.

## Results and discussion

### Detection of novel sequences and a large island of foreign DNA in ovine *B. parapertussis*

Nearly all previously available genomic information about *B. parapertussis *has been based on data from the sequenced human strain. In order to discover as yet unrecognized genes and sequences that might be unique to ovine strains, we searched for novel sequences in an ovine strain of *B. parapertussis *(Bpp5) using suppression subtractive hybridization against a pool of the three sequenced *Bordetella *genomes (including the sequenced human strain of *B. parapertussis*). Of the 40 contigs discovered in Bpp5 (GenBank:DQ518927-DQ518966), four (comprising 26 of the 100 fragments originally sequenced) had closest BLASTn hits (Additional data file 1) to genes from the anaerobic bacterium *Desulfovibrio vulgaris *(DVU2019, 2022, 2025, and 2026). To explore the *B. parapertussis *chromosomal region carrying these sequences, we constructed a fosmid library from Bpp5 DNA and probed it for the *D. vulgaris*-like sequences. Complete sequencing of a 34 kb fosmid insert (from pBpp5fos495) that hybridized to these probes revealed a large region of foreign DNA immediately downstream of the locus encoding the glycine tRNA (data not shown; GenBank:DQ515909). The foreign DNA shared homology to sequences from several unrelated species, suggesting the presence of an island of mobile horizontally transferred DNA. Partial sequencing of an overlapping fosmid insert (from pBpp5fos353) indicated that this island extended for at least 50 kb in the Bpp5 genome (see Additional data file 2 for a schematic of this region). This discovery is striking given the extremely low rates of gene acquisition in other *Bordetella *species [12,13], and may indicate that ovine *B. parapertussis *strains have access to a source of new genetic material to offset the ongoing genome degradation that marks the process of host specificity in this genus.

We designed microarray elements for the 40 novel contigs obtained from Bpp5 by subtractive hybridization (see Additional data file 1 for sequences) and added them to our *Bordetella *microarray to screen other human and ovine strains for these sequences. Of these, 38 array elements yielded data passing the quality filters, and only one of these sequences was detected in any of the 28 human strains. Sixteen array elements were variably detected among the forty-three ovine *B. parapertussis *strains, while the rest were detected in all ovine strains. All the new array elements were detected in Bpp5, from which the novel sequences were originally identified, as well as in two other strains from sheep in New Zealand (Bpp3 and Bpp4), all three of which cluster tightly based on these data and their total gene contents (see Additional data file 3 for a maximum parsimony tree of all strains).

Among the 40 ovine strain-specific contigs were 5 LPS genes that differed from all of the previously sequenced *Bordetella *LPS genes. Microarray elements for these genes were detected in all 43 ovine strains. Complete sequencing of a fosmid carrying this region from Bpp5 (from *bplE *to *wbmU*; pBpp5fos64 (GenBank:DQ519081)) revealed that the Bpp5 LPS locus is colinear with the corresponding locus from *B. parapertussis *12822 (see Additional data file 4 for alignment), with one deleted gene (*wbmE*), a truncated gene (*wbmD*), and a gene with a frameshift (*wbmI*). The genes at either end of the Bpp5 locus are very similar to those from 12822 (average sequence identity of 98.7% for *bplE*-*L*, *wbmA*-*C*, *wbmF*-*H*, and *wbmQ*-*U*), while the internal genes (*wbmI*-*P*) are significantly different (65.0% average sequence identity), and the Bpp5 open reading frame located in the position of *wbmK *appears to be completely different, with the highest BLASTn hit to a probable methyl transferase from *Wolinella succinogenes *(WS2195) (Additional data file 4).

LPS is an antigenic structure that usually causes bacterial pathogens to induce a strong host immune response. A variant LPS structure in ovine *B. parapertussis *strains might reflect a distinct type of host relationship with, or reactivity for, these strains. We previously reported highly variable LPS gene content in a collection of *B. bronchiseptica *and *B. parapertussis *strains [13]. Further examination of 39 *B. bronchiseptica *strains revealed four genetic profiles and multiple LPS structures distinguishable by electrophoresis [14]. Our observation that all 43 ovine *B. parapertussis *strains hybridized to the microarray elements for the unique LPS sequences isolated from Bpp5 may indicate that the ovine *B. parapertussis *group has less variation at this locus than *B. bronchiseptica *strains.

In addition to the novel sequences found by subtractive hybridization, previous comparative genomic hybridization (CGH) results indicated the presence of a novel *fim2 *gene in three ovine strains of *B. parapertussis*, as well as 16 of 22 *B. bronchiseptica *strains [13]. This gene encodes the major subunit of serotype 2 fimbriae, which are antigens and adhesins in *Bordetella*, so we hypothesized that this *fim2 *allele might play a role in host specificity. In previously published work, two ovine *B. parapertussis *strains showed a greater level of adherence to ovine tracheal rings than did two human strains [15], and a *B. pertussis fim2 *mutant had decreased adherence to human laryngeal cells [16]. We found that DNA from *B. bronchiseptica *and ovine *B. parapertussis *strains containing the novel *fim2 *gene showed some cross-hybridization to the probe for the *B. pertussis fim2 *gene, which we then used to identify the corresponding chromosomal fragment from an ovine *B. parapertussis *strain (Bpp4).

Sequencing of this region from ovine *B. parapertussis *strain Bpp4 revealed a *fim2 *gene that has only 69% nucleotide identity and 60% amino acid identity to the *fim2 *gene in the sequenced human *B. parapertussis *strain 12822, but has 89% nucleotide identity and 88% amino acid identity to the gene from *B. pertussis *(with greater divergence at the 5' end). The novel ovine *B. parapertussis fim2 *gene is located in a different part of the genome than the previously known *fim2 *gene, between two other fimbrial genes (*fimN *and BPP1684). When a probe specific for the novel *fim2 *gene was added to the microarray, we detected the sequence in all but one of the other ovine strains and none of the human strains. It appears that at some point after the divergence of human and ovine *B. parapertussis *strains, either the ovine strains acquired the additional *fim2 *gene through a gene duplication event, or the human strains lost the second *fim2 *gene. Since most *B. bronchiseptica *strains also have the second gene, we favor the latter hypothesis. This *fim2 *allele may have been unnecessary for colonization of the human respiratory tract or may have been a liability for these strains due to the antigenicity of its protein product.

### Human and ovine strains are distinguished by stable differences in gene content

**Figure 1:**
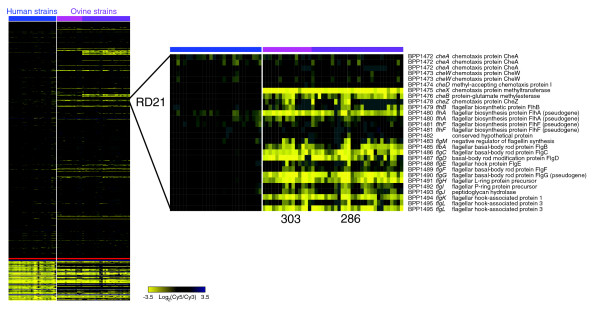
Comparative genomic hybridization of 28 human and 43 ovine strains of *B. parapertussis*. Each column represents a strain, ordered according to a maximum-parsimony tree topology. Each row represents a microarray element (usually the average of duplicates), arranged in order according to the sequenced strain 12822 (represented in the far left column), with sequences not found in that strain below the red horizontal line. Three major clades (which received >95% bootstrap support; see Additional data file 3 for tree) are denoted by colored bars. Missing data are gray (display excludes elements with missing data from more than 35% of strains). RD21 includes chemotaxis and flagella genes that are not detected in ovine strains. In some cases, data for multiple different array elements for the same gene are shown.

A maximum parsimony analysis of the data from CGH of 28 human *B. parapertussis *strains and 43 ovine strains revealed a complete separation between human and ovine strains, and two distinct subgroups within the ovine strains (Figure 1 and maximum parsimony tree in Additional data file 3). In addition, three *B. bronchiseptica *strains representing the major clades identified previously by CGH-based phylogenetic analysis [13] were included in the maximum parsimony analysis. In agreement with our earlier CGH-based phylogeny [13], the two host-restricted groups of *B. parapertussis *appear to be sister clades descended from an unidentified common ancestor of unknown host range. These results differ from the evolutionary relationships deduced using data from multilocus enzyme electrophoresis [17] or multilocus sequence typing [14], which indicated that human and ovine strains of *B. parapertussis *evolved independently from separate *B. bronchiseptica*-like ancestors. The CGH-based phylogeny, which supports the distinction of a monophyletic *B. parapertussis *clade from *B. bronchiseptica*, is based on the largest number of variable genomic features, and includes significantly more *B. parapertussis *strains of both human and ovine origin than previous analyses. However, several assumptions have been made in this analysis that may influence the inferred phylogeny. In addition to the caveats accompanying the evolutionary model of unidirectional gene loss (discussed in [13]), it is also possible that the gene complements of human and ovine strains of *B. parapertussis *have been subjected to similar selective pressures for gene loss or retention that have resulted in the two clades appearing more closely related by CGH-based analysis than they would in a phylogeny based on features that are more neutral with respect to fitness, such as silent nucleotide substitution.

There were a total of 57 regions of difference (RDs) among the 71 *B. parapertussis *strains, which consisted of 654 array elements (11.7% of the superset of array elements detected in any strain). An additional 52 array elements were designated 'single gene variants', as they were not part of a block of contiguous variable genes. Three of the RDs (RDs 47, 55, and 57) contained phage genes that had variable hybridization intensities in many strains, and seven RDs were not detected in some strains from each host (RDs 4, 12, 23, 27, 29, 38, 56). Of the remainder, 13 RDs included genes detected in the majority of the ovine strains but not human strains, and 34 RDs contained genes that were detected in most of the human but not ovine strains. This difference in number may reflect the bias in the design of the original microarray, which was based on the three sequenced *Bordetella *strains, one of which was a human *B. parapertussis *strain. When 38 array elements representing novel sequences found in an ovine strain were added, none were detected in any human strain (see above). The human *B. parapertussis *strains showed very little variation in gene content, with no RDs detected within the group, despite being collected from patients in nine countries over several decades. This similarity in gene content is in agreement with our previous study on a more limited strain collection [13], and with other typing methods [9-11].

Several functional categories of genes were under-represented among the RDs, including ribosome constituents, macromolecule synthesis/modification, biosynthesis of cofactors and carriers, energy metabolism, and cell envelope. Over-represented categories included degradation of small molecules and chemotaxis/motility genes; the latter comprised chemotaxis protein genes *cheB *and *cheX*-*Z*, the flagella genes *flhA*, *B*, *F *and *flgB*-*M *(RD21; Figure 1), and the *B. bronchiseptica pilT *gene (not found in the sequenced strain of *B. parapertussis*), which shares approximately 70% amino acid similarity with twitching motility proteins in *Pseudomonas aeruginosa *and *Xanthomonas axonopodis*. No *B. parapertussis *strains have been found to be consistently motile *in vitro*, so the significance of these genes is not known, although it is possible that motility is restricted to settings within the host. In addition, laterally acquired elements (phage genes) and proteins with no known homologs were over-represented in the RDs.

Within the 43 ovine strains, two subgroups of strains were distinguished by 9 RDs (108 array elements). These subgroups consisted of 15 and 28 strains, and did not appear to be correlated with country of isolation, growth on tyrosine agar, or citrate utilization (data not shown). Only a single small RD (RD38) varied among the ovine isolates within these subgroups. This relatively small amount of gene content variation between ovine *B. parapertussis *strains was unexpectedly low, since this strain collection was significantly larger than other collections that were reported to include more variation, as assessed by different methods. It is possible that each ovine strain carries large unique regions of DNA that are not represented on our microarray, but we find this unlikely because all the ovine strains surveyed shared the novel genes detected by subtractive hybridization of a single ovine strain. These data suggest that ovine strains have fairly stable genomes, containing significantly more genetic material than is currently appreciated.

### Transcriptional profiling reveals significant differences between human and ovine strains

Transcript abundance patterns for two human strains and two ovine strains grown under standard laboratory conditions showed significant differences between the host-restricted groups, even after excluding genes known to be missing from the strains. Of the 155 transcripts whose relative abundance was found to vary significantly between human and ovine strains (represented by 171 microarray elements; false discovery rate = 0.96%), 135 had lower abundance in the ovine strains. Among these were transcripts for the LPS genes *wbmF *and *wbmH*. Ovine strains also had lower levels of transcripts for the virulence-associated genes encoding tracheal colonization factor (*tcfA*), dermonecrotic toxin (*dnt*), and outer membrane porin protein Q (*ompQ*), as well as several transcripts for genes in the *cya *locus, which encodes adenylate cyclase toxin and its secretion apparatus. Although these data cannot be presumed to describe *in vivo *gene expression, they indicate that several important toxins and other virulence factors may be regulated differently in human and ovine *B. parapertussis*.

**Figure 2:**
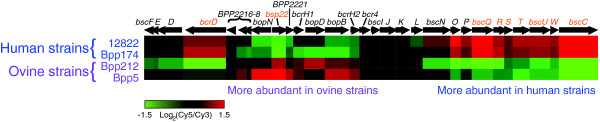
Differences in *bsc *type III secretion locus transcript abundance between human and ovine *B. parapertussis*. The sequenced strain 12822 gene arrangement is shown. Data are mean-centered for each array element. Transcript abundance of genes in orange was significantly different between the two groups according to significance analysis of microarrays (see text).

Differential transcript abundance was also detected at the *bsc *locus (Figure 2), which encodes a type III secretion system that is important for long-term tracheal colonization by *B. bronchiseptica *in rodent models of infection [18,19]. Although *in vivo *studies of type III secretion have not been conducted using *B. parapertussis*, the type III secretion-associated phenotypes were observed in ovine but not human strains *in vitro *[20]. Interestingly, we detected lower transcript levels for several putative components of the secretion apparatus (*bscO*-*W *and *bscC*) in ovine strains, but higher levels of the transcript for the secreted protein Bsp22 (as well as transcripts for secreted proteins BopB, D, and N, to lesser degrees) in these strains (Figure 2). These data support the hypothesis that type III secretion is regulated differently between host-restricted members of the *Bordetella *genus, possibly at a post-transcriptional level. Type III secretion underlies one of the most intimate interactions between host and pathogen, and may reflect co-evolution of the two partners. Precise adaptation of these secretion systems to the specific host species may be essential for proper pathogen control of host responses. Indeed, the *B. bronchiseptica *type III secretion system appears to interact with both the innate and adaptive immune systems, modulating a delicate balance and facilitating persistent infection [21].

### Differential persistence of, and inflammatory responses to, human and ovine *B. parapertussis *strains in sheep

**Figure 3:**
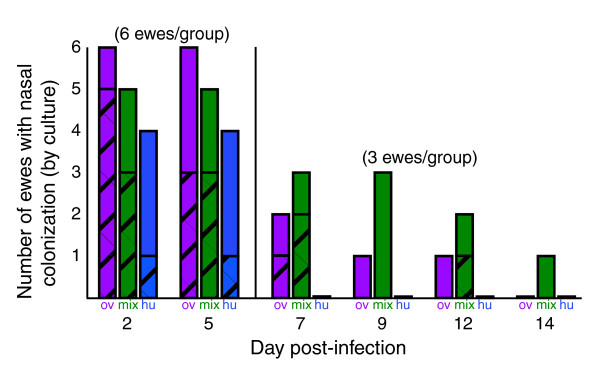
Persistence of human and ovine *B. parapertussis *in the sheep nasal cavity. Bars represent the number of sheep in each of three inoculation groups with positive nasal swab cultures of *B. parapertussis *(ov, ovine strain; hu, human strains; mix, 200:1 mixture of human and ovine strain). Plain bars indicate animals with low bacterial loads (<100 colonies); hatched bars indicate those with high bacterial loads. Half the animals were sacrificed at day 7 and half at day 14 post-inoculation.

Previous experimental inoculations with *B. parapertussis *have been conducted in mice [22,23], and in lambs using intratracheal inoculation [24,25]. To simulate the dynamics of natural infection more accurately, we inoculated adult ewes intranasally with a large dose of either the sequenced human strain of *B. parapertussis *(12822), an ovine strain (Bpp5), or media alone. In addition, we inoculated sheep with a 200:1 mixture of the human and ovine strains to observe the effects of a competitive infection. Nasal persistence (monitored by nasal swabbing three times per week) of the ovine strain group showed a steady decline over the 14 days of the experiment, while the human strain was not detected at as high a level by 2 days post-inoculation, and was cleared from all animals by 7 days post-inoculation (Figure 3). Remarkably, almost all the colonies recovered from the co-inoculated animals throughout the experiment were identified as the ovine strain (337 of 346 colonies, 97%), despite an inoculation ratio of 200:1 in favor of the human strain, indicating that the ovine strain was able to outcompete the human strain during the initial stages of infection and achieve a similar level of colonization as when the ovine strain was inoculated alone. This suggests that the human strain did not block access to components in the host required for the ovine strain to persist and multiply.

**Figure 4:**
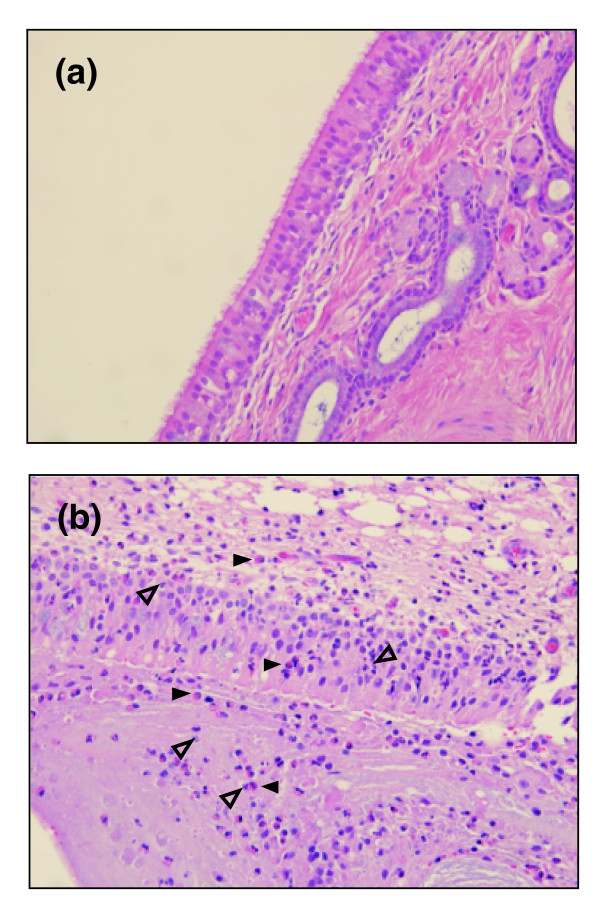
Representative sections of nasal mucosa fromewes 14 days post-inoculation with ovine or human *B. parapertussis*. (Hematoxylin and eosin stain, 400× magnification.) **(a) **Ewe inoculated with ovine strain. The epithelium has intact epithelial cells. The section lackssignificant infiltrates of inflammatory cells. **(b) **Ewe inoculated with human strain. The epithelium is covered by mucinous materialadmixed with neutrophils, eosinophils, necrotic cell debris, and seroproteinaceous fluid. Within the epithelium and lamina propria are moderate infiltrates of neutrophils (open arrowheads)and eosinophils (filled arrowheads). The lamina propria is moderately expanded by edema.

Upon sacrifice of the animals at day 7 or 14 post-inoculation, we observed a higher degree of granulocytic infiltrate in the nasal turbinates of the group infected with the human strain (Kruskall-Wallis test, p = 0.15 for both days combined; statistical power was limited due to small sample sizes) compared to the other groups (Figure 4). We also observed a lower level of lymphocyte infiltrate in the ovine strain group (p < 0.04).

The stronger immune response elicited by the human strain may have led to its earlier clearance, although other factors, such as lesser ability to adhere to host cells, may be involved as well. A previous study reported only a mild neutrophil infiltration in bronchoalveolar lavage fluid taken one day after intratracheal inoculation of lambs with ovine *B. parapertussis*, but this infiltrate had largely disappeared by day 3 [24]. In addition to neutrophils, we also observed a greater degree of eosinophil infiltrate in nasal turbinates from the animals inoculated with the human strain of *B. parapertussis *(Figure 4). Although eosinophils typically are associated with parasitic infections, their migration appears to be mediated by LPS [26]. The different LPS gene content between human and ovine *B. parapertussis *might reflect LPS structures that vary in their ability to stimulate eosinophil migration into the sheep respiratory tract.

No *B. parapertussis *was detected by culture in the nasal turbinate, trachea, tracheobronchial lymph nodes, lungs, or bronchoalveolar lavage fluid of most animals after sacrifice, although a small number of colonies were isolated from the turbinates of one ewe inoculated with the ovine strain and one with the mixed strains (at day 7 and 12, respectively), the lung of one ewe inoculated with the human strain (at day 7), and the bronchoalveolar lavage fluid of one ewe inoculated with the ovine strain (at day 7). There were no significant differences in the levels of macrophage infiltration, necrosis, or cilia loss in the nasal turbinates, tracheas, tracheobronchial lymph nodes, or lungs taken from the different groups of sheep. All the sheep inoculated with *B. parapertussis *exhibited no outward signs of disease, and most showed only minor histopathological effects. This mild pathology is consistent with previous experimental inoculations of lambs with ovine *B. parapertussis *[24,25].

## Conclusion

Overall, the results described here suggest that host specificity of *B. parapertussis *results from a process of host-pathogen co-evolution, characterized by the adaptation of structures and systems at the host-pathogen interface, such as LPS, fimbriae, and type III secretion, as well as alterations in regulation of toxins and other virulence-associated genes. These kinds of changes may account for our finding that a host species-adapted strain produced a less dramatic host immune response and was less effectively cleared by the host.

LPS may be a common factor in host-species adaptation of bacterial pathogens. LPS variation appears to play a role in the host adaptation of *Salmonella*, in which competitive exclusion of strains with the same O-antigen is hypothesized to contribute to the establishment of a single dominant serotype in each host population [27]. The significance of LPS variation among *Bordetella *species is not fully understood, but O-antigen mutants of *B. bronchiseptica *and human *B. parapertussis *were found to be defective in colonization in mice compared to wild type, and the magnitude of the defects differed between the species [28].

The relatively large extent of transcriptional differences between human and ovine strains of *B. parapertussis*, in comparison to the extent of gene content differences detected by comparative genomic hybridization, may shed light on adaptation in other species with limited genetic diversity. For example, in both *Mycobacterium *and *Brucella*, species with different host preferences appear to have similar gene content and chromosomal organization [29-31], and transcriptional profiling might produce further insights into host-species adaptation of these genera.

## Materials and methods

### Bacterial strains and growth conditions

*Bordetella *strains used in this study are listed in Additional data file 5. Strains individually discussed in this report are: *B. pertussis *Tohama I, isolated in Japan in the 1950s; *B. parapertussis *12822, isolated from a human in the United States in the 1940s; and *B. bronchiseptica *RB50, isolated from a rabbit in the United States in the 1990s. These strains were sequenced at the Sanger Centre [12]. *B. parapertussis *strains Bpp4 and Bpp5 were both isolated from sheep in New Zealand, and Bpp212 from a sheep in Scotland. Bpp174 was isolated from a human in Sweden. Strains were grown in a liquid suspension using modified Stainer-Scholte media (SSM) at 37°C with shaking. Sheep nasal swabs and tissue homogenates were plated on Moredun *Bordetella *medium (MBM) plates, which are selective for *B. parapertussis *[32], and incubated at 37°C for 4 to 5 days.

### Subtractive hybridization

Suppression subtractive hybridization was performed using the PCR-Select Bacterial Genome Subtraction kit (Clontech, Mountain View, CA, USA), with modifications as previously described [13]. The tester DNA was from Bpp5, an ovine *B. parapertussis *strain isolated in New Zealand. The driver DNA was a pool of equimolar amounts of genomic DNA from the sequenced strains *B. pertussis *Tohama I, *B. parapertussis *12822, and *B. bronchiseptica *RB50. Four hundred and eighty fragments were recovered, cloned, re-amplified, and printed on microarrays. The microarrays were cohybridized with DNA from the tester strain and the driver pool, and the 100 spots with the highest ratios of signal from tester versus driver were chosen for sequencing. Sequence from 94 SSH products was of sufficient quality to analyze, and 71 of these were found to overlap with at least one other product. Assembly of the sequences produced a total of 40 nonoverlapping contigs composed of one or more SSH products (GenBank:DQ518927-DQ518966). In a previous control experiment, approximately 70% of sequences unique to the tester pool were recovered using this technique [33].

A fosmid library of Bpp5 DNA was constructed using the CopyControl fosmid library production kit (Epicentre, Madison, WI, USA). Clones with approximately 40 kb inserts were screened by PCR using primers for sequences of interest and sequenced either partially, using primers flanking the insert, or completely, by shotgun sequencing.

### Supplementation of *Bordetella *microarray with Bpp5 sequences

Microarray elements were designed for the 40 unique, nonoverlapping subtractive hybridization products from the ovine strain Bpp5 (sequences available in Additional data file 1). PCR products for the new array elements were added to the DNA reference at equimolar concentration to the three sequenced *Bordetella *genomes.

### Comparative genomic hybridization

Genomic DNA was prepared and labeled as previously described [33]. DNA from each strain was cohybridized with a reference pool to *Bordetella *microarrays and microarray data were analyzed as described [33]. The threshold for calling an array element 'not detected' was determined individually for each array based on its distribution of log ratios, as previously described [13], except that histograms were created with a bin size of 0.14, and the first bin to the left of the main peak that had fewer than 0.65% of the total measurements was used as the cutoff. When this method was applied to the CGH data for the sequenced strain 12822, the detection sensitivity was 96.8% and specificity was 99.4%, calculated as previously described [13]. Phylogenetic analysis was performed essentially as described previously [13]. The dataset was recoded in binary representation (array element not detected = 0, detected = 1), and maximum-parsimony analysis was performed using PAUP 4.0 (Sinauer Associates, Sunderland, MA, USA), with evolutionary assumptions as described. The analysis used a heuristic search with 100 replicates of random sequence addition, and 100 bootstrap replicates were performed, each with 100 iterations of random sequence addition. RDs were defined as two or more adjacent array elements (separated by a maximum gap of one probe) not detected in at least four (of 71) strains. Single gene variants were defined as one array element not detected in at least 10 strains. Array elements in RDs were analyzed by functional category ([34], as classified by [12]) using binomial distribution with Bonferroni correction (results reported are significant at p < 0.05). Primary data have been deposited (ArrayExpress:E-TABM-94, E-MEXP-399) [35], and files with RDs marked and binary recoding of data in pre-clustering (PCL) format [39] are available in Additional data files 6 and 7.

### Analysis of transcript abundance profiles

Mid-log phase cultures were diluted in fresh SSM to a calculated absorbance of 0.05 at 600 nm. Each strain was grown in duplicate. When cultures reached mid-log phase (OD_600 _1.0 to 2.0), cells were collected and RNA was isolated, labeled, and hybridized to microarrays as described [33]. Data have been deposited (ArrayExpress:E-TABM-95) [35]. All microarray elements from CGH RDs that were not detected in any of the strains used for transcript abundance profiling were excluded from the analysis, duplicate cultures were averaged, and significance analysis of microarrays [36] was performed using all spots with data in all strains.

### Isolation and sequencing of novel *fim2 *gene

A Southern blot of *Not*I-digested Bpp4 genomic DNA was probed with the *B. pertussis *strain Tohama I *fim2 *microarray probe sequence. Fragments of the appropriate size were extracted and cloned, and colonies screened by colony blots to identify a clone carrying the *fim2 *sequence. The insert sequence was derived by primer walking (GenBank:DQ499949). A microarray probe specific for the new *fim2 *sequence was added to the arrays.

### Sheep inoculation with *B. parapertussis*

Liquid cultures to be used for inocula were diluted to approximately equivalent optical densities and delivered as 5 ml/nostril (10 ml/sheep). Twenty-four healthy adult female sheep (Suffolk and Suffolk-cross) were divided into four groups of six; each group was housed in a separate room. Group 1 was inoculated intranasally with 1.1 × 10^11 ^cfu ovine *B. parapertussis *(Bpp4), group 2 with 2.2 × 10^11 ^cfu human *B. parapertussis *(strain 12822), group 3 with an approximately 200:1 mixture of human and ovine *B. parapertussis *(2.2 × 10^11 ^cfu human strain and 1.1 × 10^9 ^cfu ovine strain), and group 4 with media alone. Nasal swabbing was performed one week before the inoculations, and three times a week thereafter. Calcium alginate fiber tipped aluminum applicator swabs (Fisher Scientific, Pittsburgh, PA, USA) were inserted as far into the nasal cavity as possible and rotated, then streaked heavily onto MBM plates and rinsed vigorously in 200 μl physiological salts solution (100 mM potassium acetate, 20 mM HEPES, 2.5 mM magnesium acetate). The swab rinses were boiled for 15 minutes and 1 μl of the lysate was used as template for PCR to detect IS*1001*, which is unique to *B. parapertussis*, using primers BPPA and BPPZ [37]. The same PCR was used to confirm the identity of colonies that grew on MBM plates. The PCR mix contained 1 × PCR Buffer II (Applied Biosystems, Foster City, CA, USA), 1.5 mM MgCl_2_, 0.6 mM dNTPs, 20 pmol each primer, and 1.25 U AmpliTaq DNA polymerase. The cycling conditions were 96°C for 10 minutes; 35 cycles of 95°C for 30 s, 60°C for 30 s, 72°C for 45 s; and a final extension at 72°C for 5 minutes. PCR products were run on 2% agarose gels and visualized with ethidium bromide staining. Sensitivity of detection was determined to be <100 cfu, using dilutions of known quantities of Bpp4. To distinguish between the human and ovine strain in colonies recovered from the co-inoculated animals, multiplex PCR was performed on randomly selected colonies from each sample (total of 346) with the *B. parapertussis *primers BPPA and BPPZ as well as primers specific for the *fim2 *gene found in Bpp4 but not 12822 (GTCCTCGCCTGGCACATT and GGTCGCCAACGTTCTTCAA), using the PCR conditions and cycles described above.

Half of the animals in each inoculation group were sacrificed on day 7 post-inoculation, and the other half on day 14. Samples were taken of the nasal turbinate, mid-trachea (approximately 18 cm above bifurcation), tracheobronchial lymph node, and lungs (combined right and left caudal and cranial lobes). Each tissue was weighed and homogenized in phosphate-buffered saline (PBS), and 100 μl of four serial dilutions (5- to 5000-fold) were plated in duplicate on MBM plates. In addition, the accessory lobe of each sheep was filled with 25 ml PBS and aspirated, and 100 μl of the recovered bronchoalveolar lavage fluid was plated undiluted.

Sections of tissues were scored subjectively from 0 to 4 on predetermined scales for degree of leukocyte infiltration, necrosis, and cilia loss. Leukocyte infiltration for intraluminal exudates (subdivided into granulocytes, macrophages, and lymphocytes) was scored as: 0 = no infiltration (no remarkable lesions); 1 = detectable infiltration (minimal); 2 = infiltration involving <30% of the section (mild); 3 = dense infiltration involving 30% to 60% of the section (moderate); 4 = dense infiltration involving >60% of the section (marked/severe). Extent of erosion/ulceration of respiratory epithelia was scored as: 0 = no erosion; 1 = one region; 2 = two regions; 3 = multifocal (more than three regions); 4 = greater than 90% of respiratory epithelia. Cilia loss was scored as: 0 = no loss; 1 = one focal region; 2 = two regions; 3 = multifocal (more than three regions); 4 = diffuse loss.

## Additional data files

The following additional data are included with the online version of this paper. Additional data file 1 is a table of BLAST results and microarray elements designed for the unique sequences recovered from ovine *B. parapertussis *strain Bpp5 by subtractive hybridization, as well as the new *fim2 *probes added to distinguish between Bpp5 fim2 and Bpe60 (Tohama I) *fim2*. Additional data file 2 is a schematic of a large island of foreign DNA found in *B. parapertussis *Bpp5 (ovine strain), in which highest BLASTn hits are denoted by colored bars indicating the species. The pBpp5fos495 insert was completely sequenced (indicated by the solid black bar); the pBpp5fos353 insert was sequenced only at the ends (dashed bar). Additional data file 3 shows the maximum parsimony tree of 71 *B. parapertussis *strains and 3 *B. bronchiseptica *strains, generated using recoded CGH dataset in binary representation (array element not detected = 0, detected = 1), with evolutionary assumptions as described previously [13]. Black circles indicate clades with ≥95% bootstrap support. Strain Bpp267 is incorrectly grouped due to its unusual histogram distribution, which caused the threshold for binary recoding to be artificially low, but examination of the primary data indicates that Bpp267 is very similar to the strains in the larger ovine clade. Additional data file 4 is a figure showing alignment of sequence of the LPS biosynthesis region from *B. parapertussis *12822 (human strain) with the Bpp5 (ovine strain) subtractive hybridization product Bpp5fos64, using ACT [38], in which red blocks indicate high sequence similarity. Additional data file 5 is a table listing features of strains used in this study. Additional data file 6 is a comma-separated values file in PCL format [39] containing the log ratios for all array elements. Additional data file 7 is a comma-separated values file in PCL format [39] containing binary data consisting of 'detected' or 'not detected' calls for each array element.

## Supplementary Material

Additional data file 1 BLAST results and microarray elements designed for the unique sequences recovered from ovine *B. parapertussis *strain Bpp5 by subtractive hybridization, as well as the new *fim2 *probes added to distinguish between Bpp5 fim2 and Bpe60 (Tohama I) *fim2* Click here for file

Additional data file 2 Highest BLASTn hits are denoted by colored bars indicating the species. The pBpp5fos495 insert was completely sequenced (indicated by the solid black bar); the pBpp5fos353 insert was sequenced only at the ends (dashed bar) Click here for file

Additional data file 3 The tree was generated using recoded CGH dataset in binary representation (array element not detected = 0, detected = 1), with evolutionary assumptions as described previously [13]. Black circles indicate clades with ≥95% bootstrap support. Strain Bpp267 is incorrectly grouped due to its unusual histogram distribution, which caused the threshold for binary recoding to be artificially low, but examination of the primary data indicates that Bpp267 is very similar to the strains in the larger ovine clade Click here for file

Additional data file 4 Red blocks indicate high sequence similarity Click here for file

Additional data file 5 Features of strains used in this study Click here for file

Additional data file 6 A comma-separated values file in PCL format [39] containing the log ratios for all array elements Click here for file

Additional data file 7 A comma-separated values file in PCL format [39] containing binary data consisting of 'detected' or 'not detected' calls for each array element Click here for file
